# Emotionally excited eyeblink-rate variability predicts an experience of transportation into the narrative world

**DOI:** 10.3389/fpsyg.2015.00447

**Published:** 2015-04-20

**Authors:** Ryota Nomura, Kojun Hino, Makoto Shimazu, Yingzong Liang, Takeshi Okada

**Affiliations:** ^1^Faculty of Education, The University of TokyoTokyo, Japan; ^2^College of Arts and Sciences, The University of TokyoTokyo, Japan; ^3^Graduate School of Information Science and Technology, The University of TokyoTokyo, Japan; ^4^Graduate School of Engineering, The University of TokyoTokyo, Japan

**Keywords:** eyeblink-rate variability, eyeblink synchronization, transportation, viewing experience, Rakugo, expert

## Abstract

Collective spectator communications such as oral presentations, movies, and storytelling performances are ubiquitous in human culture. This study investigated the effects of past viewing experiences and differences in expressive performance on an audience’s transportive experience into a created world of a storytelling performance. In the experiment, 60 participants (mean age = 34.12 years, *SD* = 13.18 years, range 18–63 years) were assigned to watch one of two videotaped performances that were played (1) in an orthodox way for frequent viewers and (2) in a modified way aimed at easier comprehension for first-time viewers. Eyeblink synchronization among participants was quantified by employing distance-based measurements of spike trains, *D*^spike^ and *D*^interval^ (Victor and Purpura, 1997). The results indicated that even non-familiar participants’ eyeblinks were synchronized as the story progressed and that the effect of the viewing experience on transportation was weak. Rather, the results of a multiple regression analysis demonstrated that the degrees of transportation could be predicted by a retrospectively reported humor experience and higher real-time variability (i.e., logarithmic transformed *SD*) of inter blink intervals during a performance viewing. The results are discussed from the viewpoint in which the extent of eyeblink synchronization and eyeblink-rate variability acts as an index of the inner experience of audience members.

## Introduction

Collective spectator communications such as oral presentations, movies, and storytelling performances are ubiquitous in human culture. Spectators who share time and space frequently involve their minds and bodies in fascinating performances. Some spectators would describe their experience as being ‘carried away’ by the story. This engrossing temporal experience is known as “transportation into the narrative world" (Sestir and Green, 2010). In a previous study, researchers summarized facilitators of narrative transportation (Van Laer et al., 2014). For instance, Van Laer et al. (2014, p. 803) and pointed out that stories containing more identifiable characters to audience members, plotlines that storytelling audiences can imagine, and verisimilitude all increase the likelihood that a narrative transportation will occur. In addition, an audience member’s familiarity with a story topic, attention level, transportability (i.e., “a story receiver’s chronic propensity to be transported,” see Van Laer et al., 2014, p. 804), age, education, and gender (female rather than male) all play a role in the likelihood of narrative transportation. Though these studies have focused on human traits—in other words, static factors of transportation—dynamic factors such as fluctuation between attention allocation and attention release during a performance also affect a transportive experience in live theater, as expressiveness between a performer and the audience is communicated in real-time. However, the processing mechanism by which an audience experiences transportation through the appreciation of expert performances remains a mystery.

Investigations into audiences’ transportive experience during a storytelling performance have suggested that audience attention tends to synchronize with the addition of audio-visual stimuli used during expert performances (Nomura and Okada, 2014). Nomura and Okada (2014) showed that during an expert performance, eyeblinks among participant audience members synchronized with greater frequency and more intensity compared to audience members of a novice performance, even though the expert and novice performers performed the same story. At the same time, subjective rating scores on a scale to determine transportation into the world of the story (Miall and Kuiken, 1995) including somatic responses (e.g., sweat and chills, Nomura, 2013) were much higher for participants who watched an expert performance than those who watched a novice performance. The timing of eyeblinks is interrelated within attentional process (Nakano et al., 2009; Nomura and Okada, 2014). In general, people search for a target upon which to focus their attention. If audiences find a target, they allocate their attention to it. After this focused concentration, they release their attention to prepare to search for the next target. Audience eyeblinks decrease at the moment of attention allocation while they increase at the moment of attention release. Therefore, eyeblinks tend to synchronously occur at implicit attentional breakpoints among readers while reading books (Hall, 1945) and among viewers while viewing videos (Nakano and Kitazawa, 2010). An additional qualitative analysis (Nomura and Okada, 2014, Study 2) also indicated that eyeblinks among audiences are synchronized corresponding to scene changes and high points of expressive performance. This externally coordinated attention leads to an efficient cognitive process by avoiding loss of significant information (Nakano et al., 2009). Thus, the authors concluded that eyeblink synchronization among audiences is guided by an expert performance created to make audiences comprehend the important information.

However, it is unclear how eyeblink synchronization among audiences relates to their experience of transportation. One of the possible mechanisms is that eyeblink synchronization among audiences is driven by attentional cycles, which are in turn driven by emotional processing. One’s eyeblinks usually cycle in self-paced (physiological) periods with some fluctuations. However, audience eyeblink onset might be delayed or accelerated depending on the actors’ expressions, as the timing of attention allocation and attention release are coordinated with the performance. When audience members shift their attention back and forth more frequently, in parallel with the storyline and punchlines performed by the actors, eyeblink time points vary dynamically, but sensitively, in line with the performance (Nomura and Okada, 2014). As s result, eyeblinks among audiences synchronize with each other. Because the duration of attentional cycles reflects the audience’s active involvement in a performance, durations vary more frequently than those of self-paced cycles. Such dynamic attention shifts bring audience members more emotional excitement. Their emotional excitement may motivate them to pay attention for upcoming expressions that could contain important content-related information. Thus, a reciprocal process between emotional excitement and the resulting motivated attention would affect transportative experiences. In other words, high emotional excitement and high eyeblink variability would predict that audience members experience more transportation.

The other possibility is that a situation model improves prediction accuracy and simultaneously facilitates the experience of transportation. A situation model refers to a reader’s representation of the referents and events described in a text (Van Dijk and Kintsch, 1983). More generally, it refers to a story receiver’s mental model (Johnson-Laird, 1983) using specific information that aids the comprehension of the current situation. When people comprehend a story, they construct a representation of the situation and its words and sentences (Zwaan et al., 1995). The current situation model manages new information from the aspect of temporal, spatial, or casual consistency (Radvansky and Copeland, 2001) and possibly enables people to predict the next plot twist more precisely. If an audience can construct representations of a story, they will more easily understand the meaning of the situation. In other words, a situation model reduces the cognitive burden required to comprehend a story. At the same time, this reduction facilitates the experience of transportation, because audiences can freely use remaining cognitive resources for other cognitive activities, such as focusing on the detail of expressions. Thus, a model can help an audience realize the depth of feelings expressed in a performance. While a non-experienced audience constructs a situation model by using only the knowledge accumulated through appreciation of the present story, an experienced audience constructs a model by also exploiting domain knowledge cultivated through past viewing experience. In light of this perspective, it could be predicted that the experienced audience, compared to the non-experienced audience, would gain more transportative experience from the beginning of a performance.

In summary, the mechanisms of audiences’ eyeblink synchronization reflecting the experience of transportation are as follows. On one hand, externally coordinated attention leads to dynamic eyeblink shifting, as well as emotional processing, due to which audience members are inclined to pay additional attention to the performance. On the other hand, a mental model reduces the cognitive burden of comprehending characters and plotlines of a story, while simultaneously improving the accuracy of prediction. These two mechanisms facilitate audience eyeblink synchronization. However, these mechanisms could be interdependent. In general, synchronizations caused by external inputs are possible if respective elements respond reliably to time-varying stimuli (Mainen and Sejnowski, 1995). Thus, eyeblink synchronization among audiences during Rakugo (traditional Japanese vaudeville) settings could occur owing to performance quality in addition to audience sensitivity to external stimulus. For instance, even though emotional excitement and biased distribution of eyeblinks predict the transportive experience, this result may be obtained from the experienced audiences only if domain knowledge is a necessary condition. In another case, even the non-experienced audiences may obtain a transportive experience if the performance contains sufficient information to guide their attentional process. The purpose of this study is to examine these two potential mechanisms and their relationship to each other. In the experiment, experienced and non-experienced audiences were assigned to watch one of two videos separately: an orthodox performance (played in front of frequent viewers) or a modified performance (played in front of first-time viewers) acted by the same artist (The details of the two performances will be described later). In all settings, participants’ eyeblink responses were observed.

The time cycle of inter-blink intervals (IBIs) varies when the performance contains more frequent expressions that draw the viewer’s attention, because the original (self-paced) period becomes accelerated or delayed. This leads to a higher rate of eyeblink variability. Thus, the SD of IBIs can be used as a measurement of eyeblink-rate variability on an individual level. Furthermore, emotional excitement can be measured by a subjective rating score on a humor scale, while it is no simple task to measure each audience member’s situation model *per se* during real-time processing. However, the similarity of situation models among audiences could be estimated by focusing on the reproducibility of participants’ eyeblink responses, because eyeblinks by audiences who have a common situation model would unintentionally select similar information, leading to more closely timed (i.e., more reliable) and more similar eyeblink patterns. In this study, we observed the precision of eyeblink responses by focusing on time differences within an audience, instead of defining the objective criteria or identifying audio-visual information to which an audience allocates its attention. We calculated mean eyeblink timing asynchrony and estimated mean similarity of IBI patterns between two particular audience members as group-level indices of reproducibility.

To investigate the first mechanism, we performed a multiple regression analysis with a SD of IBIs and humor ratings as an explanatory variable with a self-reported degree of transportation as a target variable. This hypothetical mechanism is rejected when SDs of IBIs or a subjective-humor response have no predictive power. Here, we were unable to eliminate the possibility that other variables suggested by previous research (Van Laer et al., 2014) were also facilitating or inhibiting the process interdependently. In the multiple regression analysis, we included age, gender, mean of IBIs calculated for each individual, knowledge of the performer (a dummy variable), and knowledge of the story (a dummy variable) as possible predicting variables. This analysis was performed across the experimental conditions, with the aim of determining whether variables of real-time processing, rather than other variables such as the nature of performance or the different viewing experiences, had a predictive power for transportive experience. As the first hypothetical mechanism was supported by multiple regression analysis, we went on to examine the second mechanism, concerning the use of a situation model. If the asynchrony of eyeblinks was lower in the experienced audience group than in the non-experienced group, it would suggest that domain knowledge had helped in the construction of a situation model. One-way ANOVA was performed to assess the interaction between viewing experience and actor expression during performance. If a situation model was unnecessary for transportation, the degree of transportation did not increase—at least in the situation in which group-level eyeblink asynchrony was high. If any other factor was suspected of contributing to the process of transportation throughout the analyses, an additional analysis was performed according to the nature of stimuli such as laughter of audience recorded during Rakugo (Japanese) vaudeville performances (i.e., *in situ*).

## Materials and Methods

### Participants

Participants included 28 males and 44 females, all native Japanese speakers. Out of 72 people who participated in the experiments, complete eye-tracking data was obtained from 60 participants (24 males and 36 females, mean age = 34.12 years, range = 18–63 years). Eye-movement data from two people was not usable due to drooping eyelids. Data from 10 other people were unusable because troubles with the instruments caused a loss of eye-detecting information leading to insufficient records. The experimenter defined participants who had viewed the type of storytelling performance used in the study more than 10 times in any situation, including through other media and live performances, as an experienced audience member. The experimenter adopted the criteria because the mean number of viewing times was usually three or four times in the daily lives of most Japanese. This meant that individuals who met the criteria seemed to seek opportunities to view Rakugo more than five times. As a result, 30 (15 experienced and 15 non-experienced) participants were assigned the videotaped performance as first-time viewers and the remaining 30 participants (15 experienced and 15 non-experienced) were assigned the videotaped performance as frequent viewers.

### The Storytelling Artist and Stimuli

In the current study, the authors asked professional Rakugo artist Kokontei Bungiku (34-year-old performer with 10 years’ experience) to record his performances. Rakugo is a traditional Japanese comic vaudeville storytelling performance in which one artist plays many characters. The stage setting is usually just a square cushion (*zabuton*) on which the performer sits to tell passed-down and newly created stories. The artist uses a Japanese fan and a traditional hand towel to represent all stage properties such as chopsticks and a sword (*katana*). In a traditional Rakugo apprenticeship of the Association of Rakugo (General Incorporated Associations), the title of first-rank performer (“Shin’uchi”) was given to Bungiku earlier than the 28 senior performers. We therefore assumed that Bungiku possesses the skills to modify his performance according to the nature of the audience. Two storytelling performances as well as the audio-visual information during the performance were videotaped. In both performances, the story Bungiku told was called “Nibansenji,” literally meaning the second brew of tea or decoction, which is semantically transferred to mean that things become a pale imitation. The outline of the story is as follows: five civilians go around the city of Edo (the old name of Tokyo) to prevent fires on a very cold winter night. After enduring the cold, they go back to a hut and have a warm meal, while passing around a small cup of warmed sake, conveniently concealed as “senji-kusuri” (decoction). Suddenly, a samurai who supervises the fire-prevention activities comes to the hut and calls for the door to be opened. Although civilians hurriedly hide the meal and sake, the samurai notices them quickly and wants to make them his own, relying on his authority. While the samurai wants another decoction (i.e., sake), one of the civilians answers that they have no more decoction. As the last line, the samurai orders as follows: “While I patrol around the neighborhood, brew the second decoction.”

The performances were recorded on December 6, 2013, in a Rakugo vaudeville setting that was recreated in a laboratory room at the University of Tokyo. The artist performed live in front of 31 frequent viewers and 24 first-time viewers. They (i.e., audience *in situ*) were different from participants of the current laboratory experiment. Several experimenters and assistants were also present in the room. The performance for frequent viewers was acted in the style of traditional vaudeville storytelling performance in everyday theater (orthodox video). The performance for first-time viewers was played in a modified way to help first-time viewers better comprehend the content of the story (modified video). The videos lasted approximately 3220 s (53 min 40 s) and 3022 s (50 min 22 s), respectively. For the first-time viewers, the artist took a few minutes to explain the traditional way of viewing this type of storytelling performance.

The videotaped performance was presented on a 19-inch monitor distanced 58 cm from each participant. The video was projected to a size of 15 cm (H) × 24.6 cm (W). The subjects’ viewing angle of the performer, who was sitting on the *zabuton*, was approximately 11.3° × 10.7° located at the center of the monitor. The projected size was approximately equivalent to the size of performers viewed by an audience seated at a 5-meter distance in the center of a vaudeville theater. The video stimuli was controlled by a desktop personal computer (Dell, Optiplex 900, CPU 3.40 Ghz, Memory 8.00 GB).

Eye movements were measured by a non-contact, eye-tracking device (EMR-AT, VOXER, nac Image Technology Inc.) at a sampling rate of 60 Hz. The eye position was smoothed using a moving-average method and recorded electronically. The eyeblinks were detected by instantaneous losses (0.3–1.0 s) of pupil with an eye-position motion that went rapidly down and then immediately up. The first time point during the detected eyeblink was identified as the onset of that eyeblink. The time duration from one onset to the next onset was defined as the IBI. Each participant’s chin and forehead were placed in fixed way on a support device to minimize the influence of head movements on eye-tracking data. Presentation of stimuli were controlled and recorded by a background program made by Visual Basic. A few time delays occurred before the presentation while the computer was loading a video. These presentation time delays were corrected using recorded time stamps.

### Questionnaire

The questionnaire package consisted of two scales (humor and transportation), two demographic characteristics (age and gender), and domain knowledge of the storytelling performance being shown. Humor as the emotional excitation in vaudeville settings was rated using a 4-point (from 1 to 4) Likert scale. The humor rating scale (Nomura and Maruno, 2011) included four items that reflected the audience’s degree of perceived humor (e.g., “I laughed or was inclined to laugh so much”). The transportive experience was rated using 18 items related to temporal transportation. Half of the items were derived from a subscale of the Literary Response Questionnaire (LRQ, Miall and Kuiken, 1995), which was translated into Japanese (Osanai and Okada, 2011). However, the wording of questions was inverted to fit into a vaudeville setting. For instance, “reading a novel” in the original text was changed to “viewing Rakugo” in the modified text (Nomura, 2013). The translated questionnaire also contained items relevant to the emotion of enjoyment in real life or items relevant to the author of the stories rather than transportation *per se* and less relevant items, which were not used. Moreover, as another aspect of the transportive experience, some items reflected subjective evaluation about participant’s own somatic responses such as sweaty palms and chills (Nomura, 2013) were included. The questionnaire asked participants to write their age and gender in the blanks on the sheet and describe their knowledge of the story and the artist in the videotaped performance. The questionnaire also asked participants to describe their impressions of the performance. In addition, participants filled out information on their familiarity with Rakugo performances by (1) using media and (2) going to the theater in their everyday lives.

### Procedure

The participants were separately invited to the laboratory room where the experimenter explained the experiment. To lessen the possibility that each participant intentionally controlled his/her eyeblink response, the experimenter withheld the actual purpose from the participants. Instead, the experimenter told the participants that the experiment “aimed to examine where you look on the monitor by measuring and recording the eye points while appreciating a Rakugo performance.” After briefly explaining the eye-tracking device, a nine-point calibration was performed. The experimenter recorded the air temperature and humidity at the starting point and checked to ensure that the videos worked well. The experimenter played a video (a muted movie of fish swimming in a group), while measuring and recording the eyeblinks of each participant as an individual frequency and asynchrony baseline within each group. Each participant was then instructed how to play the movie and the experimenter left the room. Participants started to play the assigned video on their own, while the device was measured and recorded their eyeblinks. After finishing the video presentation, the experimenter re-entered the room and asked the participants to complete the questionnaire. The experimenter explained that the actual purpose of the experiment was to measure the timings and frequency of eyeblinks while watching the storytelling performance. All participants gave permission for their eye information to be used in the study and agreed to answer the questionnaire. In addition, the experimenter asked if they had noticed that this was a study on eyeblinks. Five participants answered that they had noticed the eyeblink data, of which three were omitted from the analysis due to incomplete data (see, Participants). The other two participants suspected that the device might be related to eyeblink measurement; however, their eyeblink data were included in the analysis because they stated that they did not change the timing of their eyeblinks intentionally.

### Analysis

#### Distance-Based Analysis of Blink (Spike) Trains: Asynchrony

**FIGURE 1:**
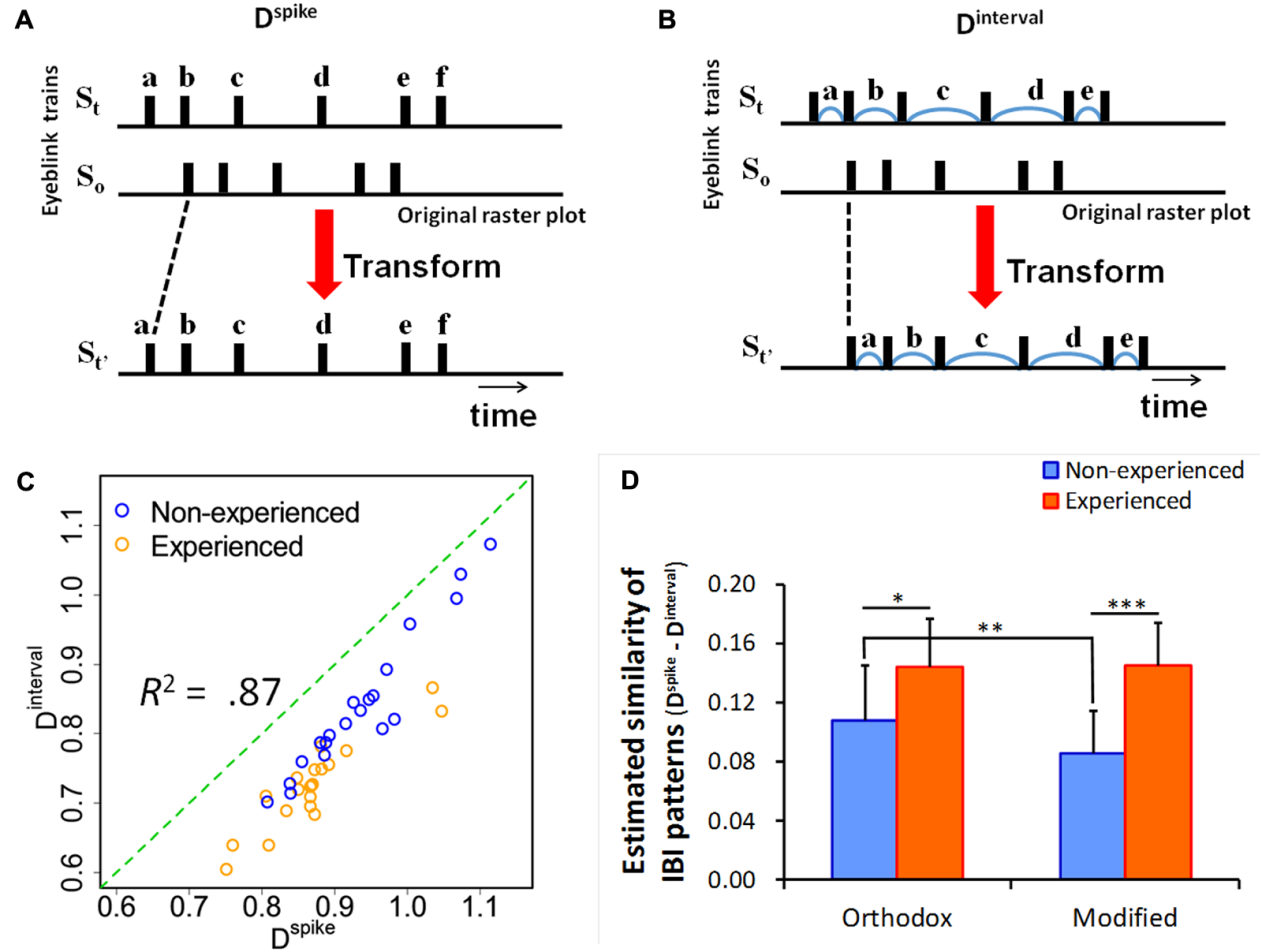
Schematic illustration of the methods to evaluate costs to transform a train to another train and estimated similarity of inter-blink interval (IBI) patterns. **(A)** The distance between the two spike trains, *S*_t_ and *S*_o_, is equal to seeking a path of the minimum cost, which transforms *S*_o_–*S*_t_, with spike times (a, b, c, d, e, f) equal to *S*_t_. **(B)** The distance between *S*_t_ and *S*_o_ is equal to seeking a path of the minimum cost, which transforms *S*_o_–*S*_t_, with IBIs (a, b, c, d, e) equal to *S*_t_. The authors originally created these two schematic illustration based on Victor and Purpura (1997). **(C)** The scatter plot of *D*^interval^ and *D*^spike^. *R*^2^ indicates the coefficient of determination. **(D)** Similarity of IBI patterns within each group estimated from the difference between *D*^interval^ and *D*^spike^. *p < 0.05, **p < 0.01, ***p < 0.001.

Victor and Purpura (1997) proposed methods to quantify the asynchrony of two particular spike trains (e.g., time series of intermittently firing neurons) focusing on the difference of spike timings. *D*^spike^ and *D*^interval^ equally evaluate the distances of two different blinking trains (**Figure 1**). However, only *D*^spike^ calculates the distance at each time point of the spikes. In contrast, *D*^interval^ takes into account the intervals of spike-by-spike. While these methods have been developed with the aim of analyzing asynchrony in firing neuron spike trains, they can be used to quantify the degree of asynchrony of particular blink trains.

*D*^spike^ is sensitive to inter-spike intervals. In contrast, *D*^interval^ is sensitive to temporal spike patterns. Although a *D*^interval^ value is constantly equal to or smaller than that of *D*^spike^, there is no difference between the values of these indices if a particular temporal pattern is started at the same time. However, the value of *D*^interval^ becomes smaller than that of *D*^spike^ when spike trains exhibit the same temporal pattern (motif) with a time delay in each time train (Victor and Purpura, 1997). Thus, the differences between these indices represent the degrees of pattern formation of IBIs. In other words, the difference in the value of *D*^interval^ compared with that of *D*^spike^ suggests the ratio explained by the pattern similarity. If the viewing experience influences a situation model constructed through a viewing performance, a significant difference of pattern similarity will be found between experienced and non-experienced audiences.

In this study, the analysis unit was set to 250 ms because a blink usually occurs at an interval elapse of least 300 ms due to physiological limits (Nakano et al., 2009). That is, the whole video recording was divided into huge numbers of time windows (i.e., bins), each of which with a length of 250 ms, and the distance was counted based on the number of bins. To evaluate asynchrony of each scene during the performance, time trains of 5 min of performance time each (i.e., 1200 units = 4 bins/s × 60 s × 5 min) were used for calculation. As the total length was different with each video, the last 50 min of video footage was accurately divided into 10 scenes (i.e., each scene containing 5 min of footage). The rest (i.e., the first 22 s in the video for frequent viewers and 220 s in the video for first-time viewers) was excluded from the time-series analysis.

All values of *D*^spike^ and *D*^interval^ were calculated using a modified version of a program provided by Victor and Purpura (1997). The program was mainly developed in the Matlab and Visual C++ environment. All *p*-values were two sided and a *p*-value of 0.05 or less was assumed statistically significant. All statistical analyses were performed using EZR (Easy R, Saitama Medical Center, Jichi Medical University; Kanda, 2013), which is a graphical interface for R (The R Foundation for Statistical Computing, Vienna, Austria, version 3.0.2).

#### Detecting the Onset of Laughter Elicited In *Situ*

To detect laughter, videos recorded in 30 frames per second were coded using ELAN 4.5.1 (Max Planck Institute for Psycholinguistics, Nijmegen), which has been developed for analyzing discourse processes and interactions among small-group members in face-to-face communication. The period of laughter was detected in the frame as the smallest unit (33.4 Hz) using only the sounds of the video. Each first frame was set as the onset of that laughter. A researcher trained in the methods of psychological study performed the coding procedure.

## Results

### Operational Checks

#### Laboratory Environment and Time Delay of Stimuli Presentation

No difference in the degree of laboratory humidity was found among the groups. Differences of time delays among the groups were not significant (range 501 ± 88.69 ms).

#### Audience Knowledge about the Performer and the Story

None of the non-experienced participants knew either the performer or the story. On the other hand, approximately a half of the experienced participants knew the performer (the orthodox performance: 46.67% and the modified performance: 33.33%) and the story (the orthodox performance: 66.67% and the modified performance: 46.67%; **Table 1**).

**Table 1:** Percentages of participants with knowledge of the performer and the story.

	Experienced participants	Non-experienced participants
**Knowledge in advance of the performer**
Orthodox performance	46.67	0.00
Modified performance	33.33	0.00
**Knowledge in advance of the story**
Orthodox performance	66.67	0.00
Modified performance	46.67	0.00

*n* = 15 for each group.

#### Reliabilities of Scales

The α coefficients of the scales were 0.77 and 0.91 for humor and experience of transportive experience, respectively. The coefficients were high enough for the following analysis.

#### Baseline of Asynchrony

To confirm that there was no difference in the total count of eyeblinks per time between groups, ANOVA was used for the IBI expressions of performer and viewing experience of the audience. The results showed no main effect and no interaction. Thus, the total rates or total numbers of eyeblinks were not different among the groups. This result was supported even if the participant’s age—a factor that may have influenced the total numbers of eyeblinks—was taken into account. Under the baseline condition, only the main effect of audience viewing experience was significant [*F*(1,338) = 9.84, *p* < 0.01]. The experienced audience value of *D*^interval^ was lower than that of the non-experienced audience (0.62 vs. 0.70, *p* < 0.05 for orthodox video and 0.60 vs. 0.71, *p* < 0.10 for modified video, respectively). This fact may indicate that experienced audience members slightly tend to synchronize their eyeblinks even when they are watching a video unrelated to domain knowledge (silent movie of a group of fish). Owing to this result, in Section “Temporal Pattern of *D*^interval^,” differences between the value of *D*^interval^ for experienced and that for non-experienced audiences were accepted only when the effect size of this comparison exceeded that of the baseline, and statistical values were significant. The values of asynchrony under the baseline condition in each group were relatively lower than those during video screening. Because the stimulus used in the baseline condition contains only visual information, the timing of the allocation would converge. On the other hand, storytelling performances included audio-visual stimulus requiring participants to integrate multimodal information.

### Multiple Regression Analysis

The mean and SD of IBIs followed logarithmic normal distribution according to the nature-of-time relevant variable. In the following analysis, logarithmic-transformed mean and SDs of IBIs were used. To examine the relationships between variables, zero-order correlations were calculated (**Table 2**). The coefficient of correlation between transportive experience and humor was very high (*r* = 0.772, *p* < 0.001). Knowledge of the performer positively correlated with transportive experience and humor. The *SD* of IBIs did not have a salient correlation with other variables.

**Table 2:** Zero-order correlation coefficients between variables used for multiple regression analysis.

	2	3	4	5	6
1. Transportation	0.772^∗∗∗^	0.071	0.197^†^	0.262^∗^	0.107
2. Humor	-	-0.008	-0.014	0.315^∗∗^	0.191^†^
3. Mean of inter-blink intervals (IBIs)		-	0.156	0.033	-0.028
4. SD of IBIs			-	0.085	0.105
5. Knowledge of the performer				-	0.703^∗∗∗^
6. Knowledge of the story					-

*N = 60, ^†^p < 0.10, ^∗^p < 0.05, ^∗∗^p < 0.01, ^∗∗^^∗^p < 0.001*.

In order to explore which variables predicted the experience of transportation, a multiple regression analysis was performed (**Table 3**). The results of multiple regression analysis demonstrated that humor strongly predicted the experience of transportation (β = 0.772, *p* < 0.001). *SD* of IBIs also regressed on the experience of transportation (β = 0.208, *p* < 0.05). The other variables such as age, gender, means of IBIs, and domain knowledge (the performer and the story, dummy variables) exhibited no significant effects. The zero-order correlations between the domain knowledge and transportive experience were weakened by taking the other variables into consideration. The coefficient of determination was considerably high (*R*^2^ = 0.64).

**Table 3:** Regression analysis of humor ratings, IBI statistical values, and domain knowledge of experience of transportation.

	*B*	SE	Standardized β	*t*-test
Intercept	0.362	0.726		0.499
Humor	0.503	0.056	0.772	9.064^∗∗∗^
Mean of IBIs	0.047	0.101	0.038	0.462
SD of IBIs	0.432	0.171	0.208	2.525^∗^
Knowledge of the performer	0.102	0.140	0.085	0.724
Knowledge of the story	-0.128	0.121	-0.121	-1.059

*R = 0.806, R^2^ = 0.649,^∗^p < 0.05, ^∗∗∗^p < 0.001*.

### Experience of Transportation

To reveal the effect of the performer’s expressions and audience viewing experience, the factor design was a two-way ANOVA performance (between the 2nd level; for frequent viewers and first-time viewers) × experience of audience (between the 2nd level; experienced and non-experienced). The dependent variables analyzed included the score of humor scale and the score of the transportation scale. When humor and transportation scale scores were combined, the main effects and interaction between performance and experience of audience were not significant. For the transportation scale score, the effect of experience was marginally significant, indicating that the score of the experienced audiences was very slightly higher than that of the non-experienced [*F*(1,56) = 2.963, *p* < 0.10, experienced 2.58 vs. non-experienced 2.37]. In addition, the simple main effect (corrected Bonferroni’s method) of the experienced audiences was marginally significant (0.30, SE = 0.17, *p* < 0.10, experienced 2.69 vs. non-experienced 2.39).

### Eyeblink Synchronization

#### Estimated Similarity of IBI Patterns

The difference between *D*^interval^ and *D*^spike^ indicates the similarity of the IBI patterns within two trains (**Figures 1A,B**). As the results of the two-way ANOVA (viewing experience × video) data of 10 scenes showed, the main effect of the viewing experience was significant [*F*(1,2066) = 25.38, *p* < 0.0001, **Figures 1D**). Sub-effect tests revealed that the estimated similarity in eyeblinks of the experienced audience was higher than that of the non-experienced audience in both performances (*p*< 0.001, 0.15 vs. 0.09 and *p* < 0.05, 0.14 vs. 0.11). In addition, the estimated similarity in eyeblinks during the orthodox video was higher than that of the modified video (*p* < 0.01, 0.12 vs. 0.10).

#### Temporal Pattern of D^**interval**^

**FIGURE 2:**
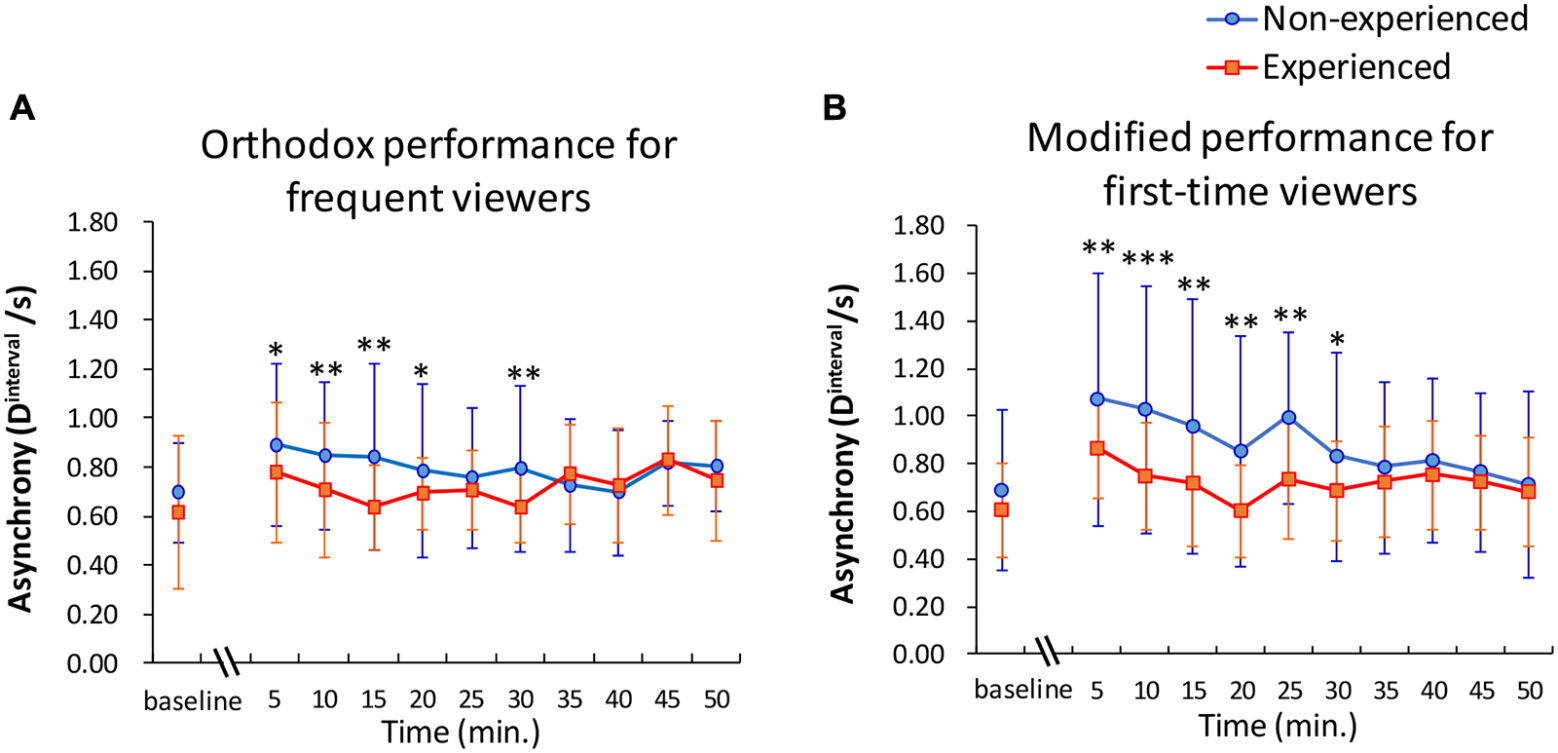
**(A,B)** Asynchrony of eyeblinks among participants at each scene (5 min) during appreciation of videotaped performance **(A)**, which is orthodox for frequent viewers, and **(B)**, which is modified for first-time viewers. Mean *D*^interval^ among all possible pairs within each group were calculated. Error bars shows the SD. Asterisks and obelisks indicate the *p*-values of *t*-tests assuming unequal variance, which were performed in each scene between experienced audience vs non-experienced audience. *P*-values corrected by the method of Bonferroni were used. ^∗^*p* < 0.05, ^∗∗^*p* < 0.01, ^∗∗∗^*p* < 0.001.

In the first six scenes (0–30 min) of each video, the average *D*^interval^ within a group of experienced participants was significantly higher than that of the non-experienced participants, with the exception of 25 min of the orthodox performance. All of the effect sizes of these comparisons exceeded those of the comparisons observed under the baseline condition. Regarding homogeneity of variances, the null hypothesis that the true ratio of variances is equal to 1 was rejected at *D*^interval^ from 15 to 30 min of the orthodox video and during all *D*^interval^ of the modified video (not shown in **Figure 2**). Overall, the index *D*^interval^ of the participants who had viewing experience remained low while watching both the orthodox video (**Figure 2A**, orange line) and the modified video (**Figure 2B**, orange line). Non-experienced participants who watched the modified performance gradually reduced eyeblink asynchrony as the story developed (**Figure 2B**, blue line). On the other hand, even non-experienced participants had reduced asynchrony as of the first few minutes to the end of the story while watching the orthodox video (**Figure 2A**, blue line). The SDs for experienced participants also stayed relatively small while SDs for non-experienced participants decreased throughout the performance.

### Effect of Laughter

**FIGURE 3:**
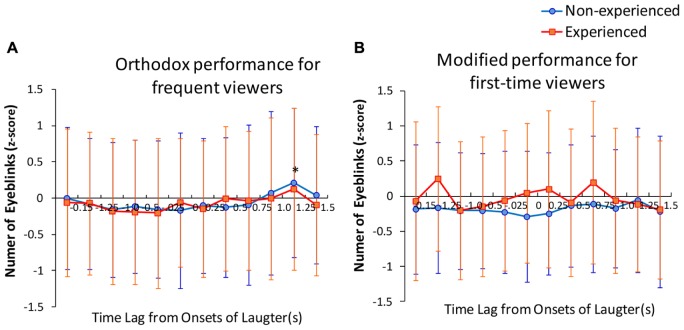
**(A,B)** *z*-scores of eyeblinks before and after onsets of laughter recorded in performance **(A)** Orthodox and **(B)** Modified. Error bars show the SD. An asterisk indicates a *p*-value of one sample *t*-test for the mean against the null hypothesis. *P*-values corrected by the method of Bonferroni were used. ^∗^*p* < 0.05.

In the case of the orthodox performance, the results of above mentioned ANOVA demonstrated relatively lower levels of *D*^interval^ for even non-experienced participants as of the beginning of the performance. However, these effects for non-experienced participants were not observed in the modified performance, possibly because of the differences between audience responses *in situ* reflecting changes in the emotional expression of the performance. In order to reveal the possible influence of laughter on the difference in *D*^interval^, the number of eyeblinks occurring at, before, and after the onset of laughter was compared. A three-way ANOVA was performed on the number of eyeblinks in each unit was normalized to a *z*-score across the performances (**Figure 3**). The factor design of this ANOVA was video (between the 2nd level; orthodox and modified) × experience of audience (between the 2nd level; experienced and non-experienced) × timing (between the 12th level; the time of six units before onset and six units after onsets of laughter). First, Mauchly’s test was conducted to check sphericity. All of the test statistics were not significant. We then used type III sum of square repeated measures ANOVA assuming sphericity. The main effects of time were significant [*F*(11,3916) = 1.853, *p* < 0.05) and none of the other main effects and interactions were significant. To identify the sub-effect, a two-way ANOVA for each video (orthodox and modified) was exerted. The results demonstrated that the main effect of timing was significant during the orthodox video [*F*(11,2420) = 2.21, *p* < 0.05). We performed one sample *t*-test for the mean against the null hypothesis (μ is 0) using a *p*-value collected by the Bonferroni’s method. Only a time point 1.25–1.50 s after the onset of laughter in the video was significantly higher than 0. All means at the other time points were not significant for rejecting the null hypothesis.

## Discussion

### Mechanisms of Transportation

Participants’ eyeblinks synchronized among the non-experienced audience at a level equivalent to that of the experienced participants through an appreciation of the performance (**Figure 2A**). As enough information seemed to be presented in each performance, even non-experienced participants appeared to be able to construct a situation model using only a temporally accumulated knowledge of the story by comprehending the storyline and the personalities of the characters. On the other hand, the SDs of the experienced participants tended to be lower than those of the non-experienced participants. This result suggests that the audience’s domain knowledge cultivated by viewing experience aids in the construct of similar situation models among the audience. The results of estimated similarity of the IBI pattern (**Figure 1C**) also suggest that experienced audiences, compared to non-experienced audiences, respond in more reproducible ways within each group. Although not all experienced participants knew the story or the performer perfectly, the experience of the participants helped to synchronize their eyeblinks. Thus, results were obtained by application of knowledge regarding typical developing patterns of storylines in the field of Rakugo performance.

However, in this experiment, the situation model supported by domain knowledge did not explain an experience of transportation fully (**Figure 1D**). The results of the ANOVA concerning transportation showed that the main effect of audience experience was weak. The results of the multiple regression analysis indicated that humor and SD of IBIs predicted a transportive experience. Other variables had no predictive effects. Van Laer et al. (2014) reveals that age, gender, and knowledge gained by education, among other variables, affects the degree of a transportive experience, based on a review of several articles (e.g., Stern, 1992; Green and Brock, 2000; Diekman and Murnen, 2004). However, the apparent effects relating to the degree of viewing experience and other demographic variables seem to be peripheral. The SD of IBIs suggests that an individual’s allocation of attention varies more frequently as he or she is inclined to predict upcoming events (Nomura and Okada, 2014). It could be said that the eyeblink-rate variability is accompanied by emotional excitement. This emotionally motivated eyeblink-rate variability might be attributed to the expressiveness of a performance and corresponding humor *in situ*. Because the same story was performed by the same performer, the differences of asynchrony must depend on the performance rather than the structure of the story. As described so far, the two mechanisms that we mentioned earlier seemed to be confirmed. It was suggested that the emotionally excited eyeblink-rate variability could be a good predictor of transportation.

The possibility of eyeblink occurrence increased at 1.25–1.50 s after the onset of laughter. This result suggests that laughter by the surrounding audiences functions as a cue for further processing (Nomura and Maruno, 2008). A time delay from the onset of laughter may be due to a time lapse between recognition and reinterpretation of a situation in the story. However, the effect was confirmed only during the orthodox performance. For non-experienced audiences, estimated pattern similarity (i.e., formation ratio of temporal patterns, “motif”) was also higher for those who watched the orthodox video than those who watched the modified video, as shown in **Figure 1D**. These results suggest that even non-experienced audiences synchronize their eyeblinks, to some extent, when appreciating a performance acted in the orthodox way usually seen in theaters. The performance that amuses experienced audiences would seem to simultaneously exert this effect on non-experienced audiences.

Although the effect of the viewing experience was confirmed, it was weak. A non-experienced audience might devote significant cognitive resources to comprehending the contents of the story, leaving very little for other resources. In contrast, an experienced audience might be engaged in a transportive experience by sparing cognitive resources in order to appreciate the details of expression, especially for an orthodox performance. The experienced audience might sometimes pay attention to a particular nuance of expression by each artist rather than simply enjoy the contents of the performance *per se*. Actually, in the free description about their impressions of the performance, some experienced audience members answered that the performer appeared to inherit the traditional style of Rakugo compared to the other performers in his generation. A viewing experience does not always lead to transportation. An implicit selection of information and a resupply of emotionally excited attention lead to a precise prediction of the next plot twist and an engrossing experience. Overall, a transportive experience would actualize under a situation in which both active leading by performance and active anticipating by the audience occur. In this sense, a performer and audience share the responsibility to create transportive enjoyment in a vaudeville setting. A performer would act as the leader in providing his/her creative expressions and the audience would play the role of actively anticipating the created world of the story.

### Dynamic Indices and Future Direction

The findings about the underlying mechanisms in real-time processing are significant in the research field of transportive experience that has focused on the traits of the receiver (see Van Laer et al., 2014). In particular, the predictive power of eyeblink-rate variability during viewing performance implies that people experience transportation through active coordination of specific external audio-visual information. Both eyeblink synchronization and eyeblink-rate variability could be useful measurements for researchers to infer the inner experience of audience members by observing unintentional behaviors objectively. The results of this study also suggest that emotional excitement motivates more attentional cognitive resources onto the actor’s expressions and the structure of the story. In this study, the positive emotion (i.e., humor) is strongly related to the transportive experience because the performance is oriented to create a sense of enjoyment or exhilaration in the audience in a vaudeville setting. However, it is not surprising if the feelings of thrill or suspense predict a transportive experience at the cinema. Future research is necessary to examine the relationships between excitement of other kinds of emotion and transportive experiences.

A possibility exists that transportation is weakened compared with that experienced through live performance because a videotaped performance cannot preserve the atmosphere *in situ*. Further study is necessary to clarify whether or not emotionally excited eyeblink-rate variability more strongly facilitates the transportive experience in real vaudeville settings. Although the humor experience was evaluated retrospectively owing to operational limitations in this study, future research will reveal the time-sequential relationships between transportation, emotional excitement, and eyeblink-rate variability by measuring ongoing physiological indices such as skin conductance, heart rate, and aspiration rhythm.

## Acknowledgments

This study was supported by JSPS KAKENHI (Grant-in-Aid for JSPS Fellows, #2408089) to RN and JSPS KAKENHI (Grant-in-Aid for Scientific Research(A), #24243062) to TO. We appreciate Kokontei Bungiku for his professional performance.
